# Assessing factors associated with changes in the numbers of birds visiting gardens in winter: Are predators partly to blame?

**DOI:** 10.1002/ece3.5702

**Published:** 2019-10-11

**Authors:** Ben Swallow, Stephen T. Buckland, Ruth King, Mike P. Toms

**Affiliations:** ^1^ Centre for Research into Ecological and Environmental Modelling School of Mathematics and Statistics University of St Andrews St Andrews UK; ^2^ School of Mathematics University of Edinburgh Edinburgh UK; ^3^ British Trust for Ornithology The Nunnery Thetford UK

**Keywords:** population change, predation, predators, songbirds, Tweedie distribution

## Abstract

The factors governing the recent declines observed in many songbirds have received much research interest, in particular whether increases of avian predators have had a negative effect on any of their prey species. In addition, further discussion has centered on whether or not the choice of model formulation has an effect on model inference. The study goal was to evaluate changes in the number of 10 songbird species in relation to a suite of environmental covariates, testing for any evidence in support of a predator effect using multiple model formulations to check for consistency in the results. We compare two different approaches to the analysis of long‐term garden bird monitoring data. The first approach models change in the prey species between 1970 and 2005 as a function of environmental covariates, including the abundance of an avian predator, while the second uses a change–change approach. Significant negative relationships were found between Eurasian Sparrowhawk *Accipiter nisus* and three of the 10 species analyzed, namely house Sparrow *Passer domesticus*, starling *Sturnus vulgaris*, and blue tit *Cyanistes caeruleus*. The results were consistent under both modeling approaches. It is not clear if this is a direct negative impact on the overall populations of these species or a behavioral response of the prey species to avoid feeding stations frequented by Sparrowhawks (which may in turn have population consequences, by reducing available resources). The species showing evidence of negative effects of Sparrowhawks were three of the four species most at risk to Sparrowhawk predation according to their prevalence in the predator's diet. The associations could be causal in nature, although in practical terms the reduction in the rate of change in numbers visiting gardens accredited to Sparrowhawks is relatively small, and so unlikely to be the main driver of observed population declines.

## Graphical abstract

We analyse data from the British Trust for Ornithology's Garden Bird Feeding Survey over a 40‐year period to determine what are the likely factors behind severe declines observed in some of the 10 species we focus on. In particular, we include abundance of Sparrowhawks, a known predator of small garden birds, to determine whether they could be a contributing factor in the declines. Our results show that they are likely to be contributing to the negative population change of their preferred prey species, although this contribution is minimal compared to other environmental factors.

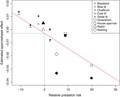

## 1 INTRODUCTION

Many species of passerine birds are exhibiting very different population trajectories across both local and national scales. Similarly, much research has shown that the ecological processes impacting birds in a garden environment are similar to those in a wider context (Toms, 2019) and analysis of such data provides scientists with the opportunity to gain a greater understanding of the potential drivers of these different trends.

Winter census counts, such as those collected through the British Trust for Ornithology (BTO) Garden Bird Feeding Survey (GBFS), suggest that different patterns, particularly those evident between urban and rural populations, are driven by different causal factors. The changes associated with agricultural intensification (Chamberlain, Fuller, Bunce, Duckworth, & Shrubb, 2000) are thought to be have been the main driver for the observed decline in some rural populations, with a reduction in the availability of favored seed during the winter months leading to decreased survival rates (Hole, 2001; Siriwardena, Baillie, & Wilson, 1999), most notably of first‐year birds (Crick, Robinson, Appleton, Clark, & Rickard, 2002). Additional evidence in support of this comes from landscape‐scale experimental work, delivering supplementary seed to winter farmland, which produced a positive effect on House Sparrow population trends on the study sites (Siriwardena et al., 2007).

The factors behind the declines seen in urban House Sparrow populations, for example, have proved far more difficult to identify and resolve, prompting significant debate. Lack of suitable nest sites (Chamberlain, Toms, Cleary‐McHarg, & Banks, 2007; Shaw, Chamberlain, & Evans, 2008), loss of invertebrate food supplies (Peach, Vincent, Fowler, & Grice, 2008), pollution (Summers‐Smith, 2007), disease, and increased predation by cats and Eurasian Sparrowhawk (hereafter Sparrowhawk) *Accipiter nisus* (Bell, Baker, Parkes, Brooke, & Chamberlain, 2010; Churcher & Lawton, 1987) have all been put forward as potential causal factors. Work by Vincent (2005), Peach et al. (2008), Peach, Mallord, Orsman, Ockendon, and Haines (2013), Peach, Sheehan, and Kirby (2014) and Peach, Mallord, Ockendon, Orsman, and Haines (2015) suggests that food availability may play a role, reducing breeding productivity and lowering postfledging survival rates. Shaw et al. (2008), working in the southwest of England, found that House Sparrows were more likely to be lost from the more affluent parts of cities, suggesting that increased demands for off‐road parking and the more managed approach to gardening reduced the habitat available to House Sparrows and, by doing so, altered foraging opportunities and predation risk. The loss of large urban gardens through development and “infilling” was found to have a negative impact on urban House Sparrow abundance (Chamberlain et al., 2007).

One area where there has been particularly vigorous debate has been around the possible role of predation in urban House Sparrow declines. For example, data collected through the BTO Common Birds Census (CBC) and the BTO/JNCC/RSPB Breeding Bird Survey (BBS) have charted a 70% decline in the House Sparrow breeding population within England since 1977 and the species has been placed on the Red List of Birds of Conservation Concern (Eaton et al., 2015; Robinson et al., 2015). Much of this debate has centered on the recovering breeding population of Sparrowhawk, a specialist predator of small birds whose English breeding population has increased by 115% since 1975 (Robinson et al., 2015). This increase in abundance, brought about through improved breeding success following a decline in the use of organochlorine pesticides (Newton & Wyllie, 1992), was accompanied by a recolonization of its former breeding range (Balmer et al., 2013) and an increased use of urban sites (Chamberlain et al., 2005). This increase spans a similar time frame to that of the declines in House Sparrows, prompting some authors to suggest a causal link (Bell et al., 2010).

Previous studies examining the potential impacts of a recovering Sparrowhawk population failed to find any large‐scale effect on breeding abundance (Newson, Rexstad, Baillie, Buckland, & Aebischer, 2010; Thomson, Green, Gregory, & Baillie, 1998). The effect of predators on their passerine prey has generally been assumed to compensate for birds that would otherwise succumb to mortality through other means, the predation being compensatory rather than additive (Newton, 1998). The lack of evidence for an effect on breeding numbers is, therefore, perhaps not surprising. Evidence for a compensatory effect may be found through the analysis of postbreeding numbers and analysis of data collected over the winter may give alternative insight into the scale of predation effects. Perrins and Geer (1980) and Newton, Dale, and Rothery (1997) studied the effect of an increase in a Sparrowhawk population on nonbreeding tits (Paridae) and other woodland species and found the seasonal pattern of mortality was altered, as was the peak in numbers. While these studies highlight local impact, they fail to provide evidence of wider scale patterns, something that can only come from a much larger study.

Isolating the impact of predation on prey populations can be challenging, and it is important to note that the method used to model the effect of environmental covariates on changes in populations of birds can also have an effect on the results. Chamberlain, Glue, and Toms (2009), Jones‐Todd, Swallow, Illian, and Toms (2017), and Bell et al. (2010) all analyzed GBFS data to test for Sparrowhawk effects on garden birds and found contrasting results. Chamberlain et al. (2009) found no significant effect while accounting for temperature and number of feeding units in the model. Bell et al. (2010), however, found significant negative effects of Sparrowhawk on House Sparrows but failed to account for any additional environmental covariates. Jones‐Todd et al. (2017) fitted multispecies spatio‐temporal models to GBFS data to jointly model changes in the predator and prey species, but once again additional environmental covariates were not modeled explicitly. In addition, the way in which we use the covariates to model change may influence our conclusions. Newson et al. (2010), for example, proposed the use of a change–change model, as opposed to a standard log‐linear model frequently used (e.g., Thomson et al., 1998), in the analysis of predation effects on songbirds. Newson et al. (2010) also failed to find any evidence that House Sparrow breeding population declines were linked to an increasing Sparrowhawk population.

The methods used in this paper aim to avoid these two problems in re‐assessing the question of Sparrowhawk effects on songbirds by using an array of possible covariates to explain the observed changes in songbird abundance, while using two alternative model formulations to test for consistency in the results.

## 2 MATERIALS AND METHODS

### 2.1 Survey methods

The study in this paper uses an extensive volunteer survey conducted annually by the BTO. The BTO GBFS has been monitoring the numbers of birds visiting private garden feeding stations each winter in the UK since 1970 (http://www.bto.org/volunteer-surveys/gbfs). For consistency of comparison with other studies, we utilize data from the onset of the study in 1970 up until 2005. GBFS sites are selected to give a representative range of garden types and spatial distribution across the United Kingdom, although they are selected from existing BTO survey volunteers (see e.g., Chamberlain et al., 2009; Chamberlain et al., 2005 for further discussion). It is therefore important to consider any subsequent analysis with the caveat that these gardens belong to observers with an existent interest in birds, and therefore may not be fully representative of the wider population. Participants note down the weekly maximum number of each species seen feeding on the provisioned food across a 26‐week period spanning the months October to March each year. Any avian predators preying on the birds visiting the feeding station are also noted. There is an average annual turnover rate of 8% for the sites and years considered in this paper, but replacement sites are selected to be similar in local habitat, size, and location to those that have left the scheme. In total over the 36 years, 693 different sites were monitored, with an average of 174 sites per year (five number summary [53, 138.5, 147.5, 226.5, 306]). This was somewhat skewed by the first 3 years, where fewer sites were monitored and after this all but 1 year saw at least 125 sites monitored. In total over the full period, 6,185 individual site‐year observations were included in the analysis.

Here, we consider the impact of a single avian predator, namely the Sparrowhawk, on the 10 species of avian prey considered by Chamberlain et al. (2009). Specifically, we model changes in Collared Dove *Streptopelia decaocto*, Blackbird *Turdus merula*, Robin *Erithacus rubecula*, Blue Tit *Cyanistes caeruleus*, Coal Tit *Periparus ater*, Great Tit *Parus major*, House Sparrow, Starling *Sturnus vulgaris*, Chaffinch *Fringilla coelebs*, and Greenfinch *Carduelis chloris*. These species are recorded regularly in the diet of the Sparrowhawk and were recorded at >80% of sites monitored and (aside from Coal Tit), >75% of all weekly observations in this analysis (Table 1).

**Table 1:** Proportion of sites where each species is observed at least once during the survey period and percentage of all conducted weekly counts where each species is observed

Species	Site occupation (%)	Mean week occurrence (%)
Collared dove	89.61	77.1
Coal tit	94.23	69.1
Starling	97.84	80.1
House sparrow	98.27	89.7
Chaffinch	98.99	83.6
Greenfinch	99.28	80.4
Great tit	99.57	85.9
Robin	99.86	90.5
Blackbird	100	87.2
Blue tit	100	96.1

**Figure 1:**
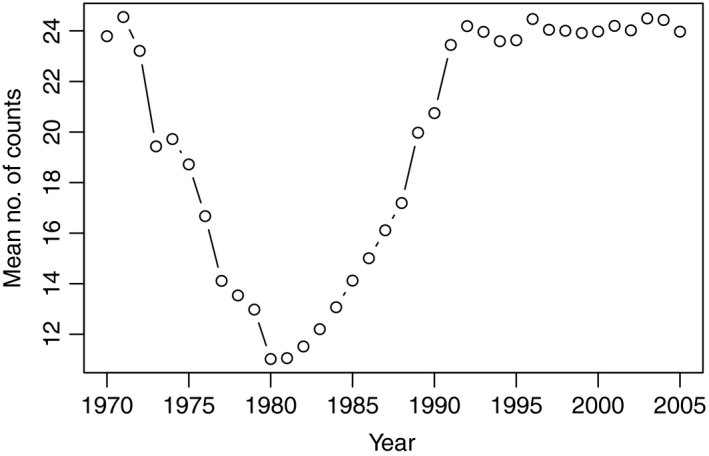
Mean number of weekly observations (of 26) completed per site in each year of the study

Figure 1 shows the variability in the mean number of the 26 weekly observations that are successfully completed each year, across sites monitored in that year. There is clearly a dip in observations during the late 1970s and early 1980s, which has been followed by much more uniform and consistent surveillance since 1990.

### 2.2 Analytical methods

Due to the fact that multiple observations are conducted within each site every year, it would require very computer intensive methods to analyze all the raw data. Following the approach adopted by Bell et al. (2010), observations across the 26 weeks were therefore averaged to give a mean of weekly maxima per site in each winter that the survey was conducted at that site. Despite the species selected being some of the most commonly recorded under the GBFS scheme, any given species is absent for many site‐by‐year combinations. This is particularly the case for more localized or habitat‐specific species such as Coal Tit, where up to 20% of the annual average site counts were exactly zero. The data therefore relate to an assumed continuous distribution, bounded below by zero, but also with a discrete mass at zero. Due to some sites being particularly large and/or well provisioned, and thus capable of sustaining large numbers of birds, the distributions are often heavily right‐skewed. Careful consideration must therefore be given to the specification of the distributional form of the fitted model.

The Tweedie distributions (Dunn & Smyth, 2005; Jørgensen, 1987, 1997) are a class of distributions belonging to the exponential dispersion models and can be specified through their mean–variance relationship. A Tweedie distribution with mean *μ* has variance $\varphi \mu^{p}$, where *φ* is a positive dispersion parameter and *p* ∉ (0, 1) a real‐valued index parameter. This class of distributions is incredibly flexible, shown particularly by the fact that they contain many standard distributions as special cases, such as normal (*p* = 0), Poisson (*p* = 2), gamma (*p* = 3), and inverse Gaussian (*p* = 4). For values of *p* ∈ (1,2), the Tweedie distributions are non‐negative continuous with a discrete mass at zero. A greater discussion of these distributions is provided in Swallow, Buckland, King, and Toms (2016), but, in essence, the distribution corresponds to a zero‐inflated distribution when *p* ∈ (1,2). The Tweedie distributions offer a unified alternative to other zero‐inflated approaches, where the proportion of zeros is commonly modeled through a Bernoulli variable, with a separate distribution for the positive observations. The power mean–variance relationship of the Tweedie distributions is also one that is common to ecological applications (Taylor, 1961). These distributions have been previously used in the environmental sciences to analyze fisheries biomass data (e.g., Candy, 2004; Foster & Bravington, 2013) and rainfall data (e.g., Dunn, 2004; Hasan & Dunn, 2010) but not for averaged data such as those analyzed in this paper.

The modeling process adopted in this paper approaches the question of Sparrowhawk effects on each of the 10 prey species independently, using two different methods. First, we use the methodology described in Swallow, Buckland, et al. (2016) and extended in Swallow, King, Buckland, and Toms (2016), modeling change in the log expected count as a function of spatially varying environmental covariates, some of which also vary over time. Let *y_it_* be the mean of weekly maxima at site *i* in year *t* for a given species of prey, then the model is expressed in the following terms:

$$y_{\mathrm{it}}∼Tw\left(\mu_{\mathrm{it}},\phi ,p\right)$$

(1) $$\log\left(\frac{\mu_{\mathrm{it}}}{\mu_{it-1}}\right)=\alpha +x_{i}^{T}\beta +v_{\mathrm{it}}^{T}\gamma +\varepsilon_{i}$$

where *x_i_* is a vector of site‐dependent covariates and *v_it_* are both site‐ and time‐dependent covariates with regression parameter vectors *β* and *γ*, respectively. We refer to this as the “standard model.” In order to make full use of the data available, *μ_i_*_0_ (i.e., the expected value at site *i* in the year before the survey commences at that site), is estimated using a data augmentation approach.

We use the following covariates that depend on site only: northing and easting values obtained from a six‐figure grid reference for each site and a two level factor variable denoting whether the site is rural (−1) or suburban/urban (+1). We also include time‐varying covariates, namely a year‐lagged expected count of the prey species being modeled to test for density dependence, the mean annual count of Collared Dove (except in the analysis of Collared Dove) and Sparrowhawk and number of ground frost days over the months the survey is conducted (October–March) from the Met Office UKCP09 gridded datasets (http://ukclimateprojections.metoffice.gov.uk/). Collared Dove is included as a “pseudo‐predator,” which has previously been used to test for spurious correlations that may arise coincidentally (Newson et al., 2010; Thomson et al., 1998). The method used to test for density dependence assumes a first‐order Markov property that the number of birds observed in year *t* depends only on the previous year, and is restricted to be negative (Dennis & Taper, 1994).

The ε_i_ denote site‐specific random effects such that

(2) $$\varepsilon_{i}∼N\left(0,\sigma^{2}\right)\;\text{for}\;i=1,\ldots ,n_{\text{site}}$$

which account for variation that is specific to the sites but unexplained by the fixed effects. Using hierarchical centering, we can reparameterize Equations 1 and 2 as follows:

(3) $$\log\left(\frac{\mu_{\mathrm{it}}}{\mu_{it-1}}\right)=v_{\mathrm{it}}^{T}\gamma +\varepsilon_{i}$$

and

(4) $$\varepsilon_{i}∼N\left(\alpha +x_{i}^{T}\beta ,\sigma^{2}\right)$$

We use this parameterization in order to reduce correlation between parameters and improve the efficiency of the parameter estimation process.

The second modeling approach, the “change model,” modifies the above methodology into the framework outlined by Newson et al. (2010), modeling change in log abundance as a function of change in covariates, that is a change‐change model. The vector of site‐specific covariates above *x_i_* remains the same. However, *v_it_*, the time‐varying covariates, are replaced with the rate of change of the log covariate between year 1 and year *t*. Under this framework the expected value at site *i* in year *t* can be written as follows:

(5) $$\mu_{\mathrm{it}}=\alpha +x_{i}^{T}\beta \;+\;\log{\left(\frac{v_{\mathrm{it}}}{v_{i1}}\right)}^{T}\gamma +\varepsilon_{i}$$

Newson et al. (2010) show an algebraic equivalence between this parameterization and one equivalent to Equation 3.

(6) $$\log\left(\frac{\mu_{\mathrm{it}}}{\mu_{it-1}}\right)=v_{\mathrm{it}}^{T}\gamma +\varepsilon_{i}.$$

The algebraic manipulation becomes a little more complicated in the presence of random effects as the new random effects *ε_i_* from Equation 6 relate to a ratio of the random effects from Equation 5. We assume that the *ε_i_* from Equation 6 is normally distributed in order to specify the model in an equivalent form to that of the first model, and can be written equivalently to Equation 4, remains.

$$\varepsilon_{i}^{\prime }∼N\left(\alpha +x_{i}^{T}\beta ,\sigma^{2}\right)$$

We adopt a fully Bayesian approach to obtaining inference on the model parameters of interest, using a Markov chain Monte Carlo (MCMC) framework to obtain samples from the marginal posterior distributions of interest. The same prior distributions are used in both modeling approaches and are specified in Table 2. Covariates are normalized to ensure all covariates are on the same scale and hence priors on the corresponding coefficients are also specified on a meaningful scale (King, Morgan, Gimenez, & Brooks, 2010). We are particularly interested in which environmental factors best explain the observed changes in populations of songbirds. We therefore include an array of environmental covariates and use reversible jump (RJ) MCMC, allowing us to quantitatively compare competing models with different covariates. The RJMCMC algorithm allows us to estimate posterior model probabilities and hence Bayes factors. In this paper we test for covariate dependence by testing the two hypotheses *θ_i_* = 0 versus $\theta_{i}\neq 0$ where *θ* ∈ {*β*,*γ*}. The common intercept α under both model specifications is assumed to be always present in the model. Kass and Raftery (1995) outline a scale for the interpretation of Bayes factors and suggest a value greater than three constitutes positive evidence in support of one hypothesis over another. We assume significant covariates to be those with Bayes factors exceeding three in relation to the above hypotheses.

**Table 2:** Prior specification for model parameters

	Parameter	Prior distribution
*β*	Regression parameters (time‐invariant covariates)	N(0,0.01)
$\gamma_{\mathrm{DD}}$	Regression parameter for density‐dependence covariate	HN(0,0.01)
$\gamma_{\left(\mathrm{DD}\right)}$	Regression parameters (for time‐variant covariates other than above)	N(0,0.01)
ϕ	Tweedie dispersion parameter	U[0,5]
*p*	Tweedie index parameter	U[0,2]
$\sigma^{2}$	Random effect variance	InvGamma (0.001,0.001)
$\mu_{i0}$	Estimated mean of year before survey starts	U[0,200]

Goodness‐of‐fit is assessed through the Bayesian *p*‐value $p_{\theta }$, a measure of posterior predictive fit (Gelman et al., 1996). The deviance is used as the discrepancy statistic and *p*‐values outside the interval 0.025 ≤ $p_{\theta }$ ≤ 0.975 would give rise to evidence of poor fit of the model.

## 3 RESULTS

The two models were run independently for all 10 species. The standard model converged very quickly. Using 20,000 iterations with the first 5,000 being discarded as burn‐in appeared to be very conservative. The change model took longer to converge and here we ran 100,000 iterations with the first 60,000 iterations discarded as burn‐in. Posterior summary statistics from the two analyses are presented in Tables 3 and 4. For the regression parameters, significance was assumed for covariates with Bayes factors >3. Goodness‐of‐fit was assessed through Bayesian *p*‐values using the deviance as the discrepancy statistic (Table 5). The only analysis giving possible concern was Coal Tit under the standard model, with an estimated *p*‐value just inside the rejection region. This may be due to a marginally bimodal distribution for this species which the Tweedie distributions have difficulty in fitting to.

**Table 3:** Posterior means (above) and Bayes factors (below) for model parameters from the standard model

Species	Intercept	Northing	Easting	Rur/sub	Density dep.	Sparrowhawk	Collared dove	Frost	ϕ	*p*	$\sigma^{2}$
Collared dove	0.0185	−0.0016	0.0099	−0.0091	−0.0466	−0.0043	NA	0.0096	0.5393	1.328	0.0129
	—	0.0705	0.2483	0.1895	**>10**	0.0515	NA	0.3194	—	—	—
Blackbird	0.011	0.0049	−0.0069	−0.0077	−0.0328	0.0103	0.0153	0.0119	0.204	1.2434	0.0032
	—	0.105	0.3951	0.9539	**>10**	**>10**	**>10**	**>10**	—	—	—
Robin	0.0044	0.0012	−0.0046	−0.0095	−0.0291	0.0049	0.003	9e−04	0.0638	1.0581	0.0013
	—	0.0292	0.4229	**>10**	**>10**	2.7092	0.1842	0.0227	—	—	—
Blue tit	−0.0205	−0.0011	−0.0044	−0.0074	−0.0142	−0.0077	−0.0017	0.0077	0.1683	1.4424	0.0019
	—	0.0309	0.1269	**3.0502**	**>10**	**>10**	0.0438	**>10**	—	—	—
Coal tit	−0.0118	0.0125	−0.0168	6e−04	−0.0243	0.0135	0.0069	0.0034	0.3323	1.265	0.004
	—	1.3283	**7.058**	0.0467	**>10**	**>10**	0.068	0.0637	—	—	—
Great tit	−0.0099	−7e−04	−0.001	−0.009	−0.0188	0	−0.0018	0.0045	0.1994	1.1796	0.0018
	—	0.0279	0.0219	**>10**	**>10**	0.0236	0.0261	0.2957	—	—	—
House sparrow	−0.0541	−0.0118	−0.0262	−0.0116	−3e−04	−0.0369	5e−04	0.0404	0.6895	1.3552	0.0147
	—	0.2492	**>10**	0.3793	0.0016	**>10**	0.0121	**>10**	—	—	—
Starling	−0.0594	−0.008	−8e−04	8e−04	−0.0014	−0.0332	0.008	0.0429	0.6982	1.3875	0.0082
	—	0.1696	0.0433	0.0378	0.0185	**>10**	**>10**	**>10**	—	—	—
Chaffinch	0.0045	0.0041	−0.0141	−0.0155	−0.0249	0.0035	0.0088	0.029	0.3815	1.3634	0.0063
	—	0.0669	**7.3963**	**>10**	**>10**	0.1306	**>10**	**>10**	—	—	—
Greenfinch	−0.0243	0.0027	−0.02	−0.0145	−0.0199	−0.0044	−0.0051	0.0104	0.5212	1.4127	0.0102
	—	0.0655	**>10**	2.4329	**>10**	0.2908	0.2192	0.374	—	—	—

Note

“—” relate to parameters always present in the model. Significant covariates are highlighted in bold.

**Table 4:** Posterior means (above) and Bayes factors (below) for model parameters from the change model

Species	Intercept	Northing	Easting	Rur/sub	S‐hawk	Collared dove	Frost	*φ*	*p*	$\sigma^{2}$
Collared dove	−0.0232	8e−04	0.0073	−0.0069	−0.0069	NA	0.0365	0.5748	1.342	0.0126
	—	0.0602	0.1451	0.12693	0.1566	NA	**>10**	—	—	—
Blackbird	0.0036	−0.0018	−0.0073	−0.0051	0.0043	0.0131	0.0076	0.2086	1.278	0.0016
	—	0.3951	**3.0556**	0.268	0.1378	**>10**	4.6705	—	—	—
Robin	2e−04	3e−04	−0.001	−5e−04	−5e−04	0.0045	2e−04	0.0681	1.0674	7e−04
	—	0.0175	0.0163	0.0204	0.0143	**3.2283**	0.0496	—	—	—
Blue tit	−0.0313	0.0027	−0.0026	−0.0015	−0.0087	0.0016	0.0087	0.1661	1.4603	0.00184
	—	0.0494	0.0406	0.0221	**>10**	0.0301	1.7093	—	—	—
Coal tit	−0.033	0.0102	−0.0146	0.0016	0.0117	0.0166	−0.0165	0.3476	1.2738	0.0034
	—	1.9351	**3.3085**	0.0282	1.2831	**>10**	**8.311**	—	—	—
Great tit	−0.02	0.0013	−0.0014	−8e−04	5e−04	0.0022	0.0069	0.2044	1.1859	0.0017
	—	0.0298	0.0306	0.0357	0.0263	0.0483	0.5576	—	—	—
House sparrow	−0.0782	0.0183	−0.0108	−0.0166	−0.0325	0.01	0.0556	0.6574	1.339	0.0105
	—	**>10**	0.4088	**>10**	**>10**	**3.3365**	**>10**	—	—	—
Starling	−0.0798	0.0076	0.0021	0.0000	−0.0187	0.0131	0.0427	0.6945	1.3864	0.0079
	—	0.2296	0.066	0.04	**>10**	**>10**	**>10**	—	—	—
Chaffinch	−0.0113	0.0083	−2e−04	−0.01	0.002	0.0122	0.0266	0.3884	1.3712	0.0061
	—	0.6088	0.0471	1.5913	0.0332	**>10**	**>10**	—	—	—
Greenfinch	−0.0393	0.007	−0.0199	−0.0131	−0.0032	−0.002	0.0182	0.5291	1.422	0.0088
	—	0.1685	>10	1.482	0.0763	0.0494	>10	—	—	—

Note

“—“relate to parameters always present in the model. Significant covariates are highlighted in bold.

**Table 5:** Bayesian *p*‐values for the two model formulations

Species	BPV (standard)	BPV (change)
Collared dove	0.8941	0.8499
Blackbird	0.8164	0.524
Robin	0.7511	0.2228
Blue tit	0.4399	0.5097
Coal tit	0.0204	0.165
Great tit	0.1683	0.2229
House sparrow	0.6263	0.5812
Starling	0.4644	0.8089
Chaffinch	0.7005	0.7182
Greenfinch	0.7132	0.8919

### 3.1 Sparrowhawk effect

Across the two models, eight of the possible 20 correlations between prey species and Sparrowhawks were found to be significant. Of these, five came from the standard model and three from the change model. Under the standard model, two of these correlations were positive and three negative. The three species showing significant correlation with Sparrowhawk under the change model were all negative and corresponded to the same three species with negative significant covariates in the standard model. No significant positive effects were found in the change model. Despite the significance of the negative effects, the results suggest at most around a 3.6% reduction, on average, in the rate of population change in response to every additional Sparrowhawk under the standard model or, equivalently, a 2.2% reduction in response to the doubling of predator numbers under the change model (interestingly both for House Sparrow).

Gotmark and Post (1996) estimated the relative predation risk for a number of species of songbirds to predation by Sparrowhawks, a measure of how frequently the species occurs in the Sparrowhawk's diet having normalized for its prevalence in the surrounding environment. In particular, species frequently found feeding on the ground have been shown to be particularly susceptible to Sparrowhawk predation (Chamberlain et al., 2009; Götmark & Andersson, 2005). Associations between high relative predation risk and negative Sparrowhawk coefficients may give further support to a genuine Sparrowhawk impact on prey populations.

**Figure 2:**
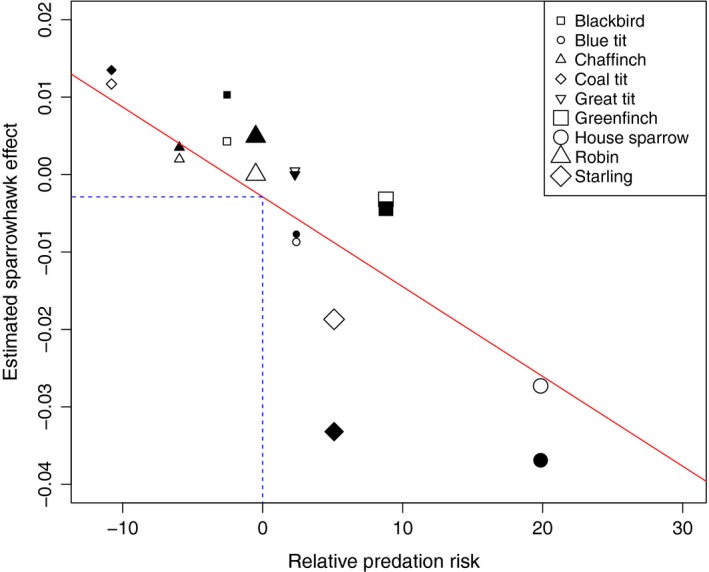
Relationship between posterior means of Sparrowhawk covariate from our standard model (filled) and change model (open) versus relative predation risk from Gotmark and Post (1996). Solid line is a fitted linear model to all the points with the dotted line showing the estimated effect when relative predation risk is zero

Relative predation risks for all of the species analyzed in this paper, aside from Collared Dove, were calculated by Gotmark and Post (1996) and following Chamberlain et al. (2009), these are plotted against the posterior means for the regression parameters obtained from the two modeling approaches used in this paper (Figure 2). A relatively strong negative relationship was found between the two measures with linear correlation estimated as −0.81, giving further support to the weak correlations found in Chamberlain et al. (2009). This suggests that those species appearing most at risk to predation by Sparrowhawks are also showing negative relationships with Sparrowhawk abundance. Of the four species most at risk from predation by Sparrowhawk, three had significant negative estimates under both our models. Under the relative predation risk, a value of zero is equivalent to a Sparrowhawk randomly searching for prey. According to the linear model fitted to our results and predation risk, a zero value of relative predation risk relates to a value close to, but slightly below, zero under the two models used in this paper. This could relate to the fact that a zero relative predation risk does not equate to zero risk and hence the prey species could still be susceptible to increases in Sparrowhawk numbers.

### 3.2 Effects of other variables

With reference to the other regression covariates, easting was found to be a more important predictor of changes in songbird numbers than was northing. Northing was only significant for one species, positive for House Sparrow in the change model. However, Coal Tit and Greenfinch were the only species with significant estimates to show consistency across the two models with respect to these two covariates, both having negative estimates for easting. The other time‐invariant covariate, the rural or suburban factor variable, showed some differences between the two models. Where significant, the parameter was always negative suggesting that urban sites are on average faring worse than their rural counterparts.

In the standard model, evidence of density dependence was found in all but two of the species, namely House Sparrow and Starling. Treating Collared Dove as a dummy predator, there were three small but significant positive correlations between prey species and Collared Dove under the standard model, and six under the change model, again all positive.

Over half of the species showed significant changes in relation to frost days. In each case the relationship was positive in the standard model. All but one of these species (Blue Tit) also showed positive relationships under the change model, with the estimate for Greenfinch and Collared Dove also now being significant. In addition, Coal Tit was the only species to show a significant negative estimate for this covariate, but only in the change model. The parameter estimates associated with this covariate were on average the largest of all the covariates, suggesting frost has the greatest effect on annual changes in the numbers of birds observed visiting gardens.

Estimates of the random effect variance *σ* ^2^ were fairly similar across model specifications, suggesting the fixed effects were capturing a similar amount of the variation in both cases. There was, however, a ten‐fold difference between the species with the smallest (Robin) and largest (Collared Dove, House Sparrow, Greenfinch) variances. Estimates of the Tweedie parameters *φ* and *p* were also consistent under both model formulations.

## 4 DISCUSSION

Our results suggest that after controlling for effects of environmental factors, such as weather and surrounding habitat, there is still an additional negative Sparrowhawk effect for three of the 10 species considered. Despite the statistical significance of the covariate for these three species, in practical terms the effect is relatively small with at most only a 3% reduction in the rate of change in observation rate attributed to each additional Sparrowhawk. Given that the majority of Sparrowhawk observations relate to single birds, occasionally two, it seems reasonable to suggest that the Sparrowhawk effect is likely a contributory factor to the declines seen in House Sparrow and Starling rather than the main cause. In addition, Blue Tit populations have remained largely stable over the UK during this period so any negative effect of Sparrowhawks appears to have been compensated for by other means.

This analysis was conducted on data relating to garden birds attracted to feeding stations. The nature of the survey protocol, as mentioned above, requires that we must be careful in extending inference from these results to wider populations of these species. Chamberlain et al. (2005) found c.96% correlation between winter abundance at GBFS sites and annual indices of relative breeding population change derived from CBC data for both House Sparrow and Starling. Due to the strong correlation in these cases, it seems reasonable to expect that the studied gardens are a good representation of wider populations. Observations of Blue Tits from GBFS were weakly negatively (−0.32) correlated with equivalent breeding density. Nonetheless, the species showing significant negative relationships with Sparrowhawks are also largely those most at risk from predation from Sparrowhawks, appearing more frequently in the Sparrowhawk's diet than would be expected based on their overall abundance (Figure 1).

The significant positive relationships of Sparrowhawk with Coal Tit and Blackbird in the standard model may well represent confounding factors that lead to Sparrowhawks recolonizing sites that were also more attractive to the former species, rather than a causal relationship. We cannot be certain that the same interpretation does not hold in reverse for the species with negative effects; however, the lack of positive effects under the change model framework and the relationship with predation risk add to the evidence for the negative effects.

Our modeling quantifies the effect of Sparrowhawks on the numbers of birds of various species attending feeding stations. It does not, however, quantify the corresponding effect on breeding populations. Many previous studies have found no evidence to suggest that Sparrowhawks have depleted the breeding densities of songbirds (Newson et al., 2010; Thomson et al., 1998). The apparent reduction in numbers observed at feeding stations when Sparrowhawks colonize a site might represent an overall depletion of the total nonbreeding population by Sparrowhawks, or might reflect a behavioral response among birds avoiding the feeding station, or spending less time at it (and hence becoming more difficult to observe). Behavioral changes, such as avoidance of sites where Sparrowhawks have been encountered previously, may lead to sublethal predation‐risk impacts, such as limited access to food and result in increased levels of starvation and a decline in population size (Cresswell, 2008; Seress, Bókony, Heszberger, & Liker, 2011). The reality may be some combination of these two explanations.

The prevalence of significant negative easting effects over northing is most likely due to the higher level of intensive agriculture seen in the eastern UK. This intensification has been shown to be closely linked to negative changes in the populations of many farmland birds (Fuller et al., 1995; Newton, 2004). This might add support to the hypothesis that the intensification of farming has led to decreases in House Sparrow populations. The decreases in farmland may be having an additional effect on the surrounding gardens in this region, something perhaps evident in the regional patterns found in House Sparrow productivity, as revealed by BTO Garden BirdWatch, another study focused on urbanized landscapes (Morrison, Robinson, Leech, Dadam, & Toms, 2014). Coal Tit is a species of coniferous woodland, that makes greater use of garden feeding stations when access to cone crops is limited by a poor seed year (Mckenzie, Petty, Toms, & Furness, 2007). The establishment and maturation of new conifer plantations may influence Coal Tit distribution and we would therefore expect there to be some pattern to the species distribution linked to easting.

The parameter relating to the categorical variable of suburban or rural habitat is, where significant, negative, suggesting that urban populations are faring less well than their rural counterparts. The negative effect of urbanization suggests that there are additional factors common to urban gardens above the level of individual site variation that cannot be explained through the other fixed effects. Previous studies on rural and urban populations confirm that different trends are taking place in the two different habitats, with populations in urban environments generally doing worse (e.g., Beckerman, Boots, & Gaston, 2007; Newson, Ockendon, Joys, Noble, & Baillie, 2009; Robinson, Siriwardena, & Crick, 2005; Vincent, 2005). The factor variable is only able to pick up general differences between the two habitat types that cannot be explained by the other covariates or the random site effects. Reasons for the significant differences in these subpopulations may warrant further study.

Our results also suggest that density dependence is an important factor governing the numbers of most species observed. It is perhaps not surprising that this is the case given that our data come from garden feeding stations, where birds may well be competing for food within a small area and where other factors linked to the densities of birds present, such as disease transmission (Lawson et al., 2012), may have a role to play.

As outlined in the methods, we included Collared Dove as a “pseudo‐predator” to act as a control alongside our investigations of Sparrowhawk effects. Our analyses revealed no negative effects of Collared Doves, but with estimated positive effects for Blackbird, Chaffinch and Starling under both models in addition to Robin, Coal Tit and House Sparrow under the change model. These correlations in numbers may indicate similar habitat or food requirements among the species.

The positive parameter estimates for frost days from the standard model are consistent with the idea that the birds are subject to a behavioral response where they move to gardens as access to natural food resources becomes restricted. Chamberlain et al. (2005) found significant negative correlations between the probability of bird occurrence and minimum temperature in all 10 species studied in this paper. The covariate used in this paper, frost days, was very strongly negatively correlated (−0.87) with minimum temperature at the same site. The negative relationship between numbers of Coal Tits visiting gardens and change in the number of frost days was contrary to the results of Chamberlain et al. (2005). The use of garden feeding stations by Coal Tits has been shown to be influenced by the size of conifer seed crops, the birds switching to feed on supplementary food more often in years with few cones than in mast years (Mckenzie et al., 2007). Access to the conifer seeds may also be influenced by winter weather conditions—the cones opening on dry days and closing on damp ones. A winter with more frost days, which are typically associated with clear skies and dry conditions, may see the cones open more often throughout the winter, leaving Coal Tits with greater access to this natural food resource and less reliant on garden feeders.

The magnitude of parameters associated with continuous covariates also gives an idea of the relative importance of each covariate in predicting the number of birds visiting the feeding sites. The climate variable used seems to be the most important on average, followed by Sparrowhawk abundance. The effect of weather, if it induces a behavioral response, is likely to be instantaneous as birds respond to a lack of food. The larger effect on counts of birds in relation to these fluctuations in temperature is therefore not too surprising.

We found a fairly large degree of variation in the consistency of species trends across sites. Robins and Blackbirds, for example, seemed to show relatively small intersite differences, while House Sparrow, Starling, Collared Dove and Greenfinch showed less consistency. This suggests that there may be additional factors affecting the latter species that we have not considered in our analyses. The species with larger random effect variances are ones that tend to form larger groups or flock together. In addition to the factors modeled, there are clearly additional factors that encourage these species to form larger groups at some feeding stations than others, such as the size of site that is not implicitly modeled here.

We see the Tweedie distributions as a widely‐applicable but underused tool in ecology and suggest they could become much more widely used outside of their current limited applications. The mean‐variance relationship has been shown to be one common to ecology, while remaining incredibly flexible and avoiding the need for strong assumptions to be made about the distribution a priori. Using the two alternative model formulations in general provided consistent results in this case. Overall neither model formulation appeared to consistently outdo the other in terms of posterior predictive power; most variation in Bayesian *p*‐values was between, rather than within, species. We propose that where possible, the use of multiple model formulations is advantageous, allowing different hypotheses and ecological mechanisms to be tested or highlighted. In addition, we have also shown that how the covariates enter a model is not arbitrary. We recommend that careful thought should always be given to the model specification to ensure it is both statistically and ecologically sensible.

## CONFLICT OF INTEREST

None declared.

## AUTHOR CONTRIBUTIONS

BS, STB, and RK conceived the ideas and designed methodology; BS analyzed the data; BS led the manuscript writing with input from MPT. All authors contributed critically to the drafts and gave final approval for publication.

## Data Availability

The cleaned data file used in this analysis has been placed on Dryad with DOI https://doi.org/10.5061/dryad.v8j1144.
